# New function for *Escherichia coli* xanthosine phophorylase (xapA): genetic and biochemical evidences on its participation in NAD^+^ salvage from nicotinamide

**DOI:** 10.1186/1471-2180-14-29

**Published:** 2014-02-08

**Authors:** Wei-Ren Dong, Cen-Cen Sun, Guan Zhu, Shi-Hua Hu, Li-Xin Xiang, Jian-Zhong Shao

**Affiliations:** 1 College of Life Sciences, Zhejiang University, Hangzhou 310058, People’s Republic of China; 2 Key Laboratory for Cell and Gene Engineering of Zhejiang Province, Hangzhou 310058, People’s Republic of China; 3 Department of Veterinary Pathobiology, College of Veterinary Medicine & Biomedical Sciences, Texas A&M University, 4467 TAMU, College Station, TX, USA

**Keywords:** Purine nucleoside phosphorylase, Nicotinamide riboside, Salvage pathway, Pyridine nucleotide cycles

## Abstract

**Background:**

In an effort to reconstitute the NAD^+^ synthetic pathway in *Escherichia coli* (*E. coli*), we produced a set of gene knockout mutants with deficiencies in previously well-defined NAD^+^*de novo* and salvage pathways. Unexpectedly, the mutant deficient in NAD^+^*de novo* and salvage pathway **I** could grow in M9/nicotinamide medium, which was contradictory to the proposed classic NAD^+^ metabolism of *E. coli*. Such *E. coli* mutagenesis assay suggested the presence of an undefined machinery to feed nicotinamide into the NAD^+^ biosynthesis. We wanted to verify whether xanthosine phophorylase (xapA) contributed to a new NAD^+^ salvage pathway from nicotinamide.

**Results:**

Additional knockout of *xapA* further slowed down the bacterial growth in M9/nicotinamide medium, whereas the complementation of *xapA* restored the growth phenotype. To further validate the new function of xapA, we cloned and expressed *E. coli* xapA as a recombinant soluble protein. Biochemical assay confirmed that xapA was capable of using nicotinamide as a substrate for nicotinamide riboside formation.

**Conclusions:**

Both the genetic and biochemical evidences indicated that xapA could convert nicotinamide to nicotinamide riboside in *E. coli*, albeit with relatively weak activity, indicating that xapA may contribute to a second NAD^+^ salvage pathway from nicotinamide. We speculate that this xapA-mediated NAD^+^ salvage pathway might be significant in some bacteria lacking NAD^+^*de novo* and NAD^+^ salvage pathway **I** or **II**, to not only use nicotinamide riboside, but also nicotinamide as precursors to synthesize NAD^+^. However, this speculation needs to be experimentally tested.

## Background

Nicotinamide adenine dinucleotide (NAD^+^) and NAD^+^ phosphate (NADP^+^) are two of the most important coenzymes in cells. They act as either electron donors or electron acceptors in more than 300 enzymatically catalyzed oxidoreductions [1,2]. NAD^+^ also plays an essential role in producing ATP, and is involved in various cellular processes as a substrate for a number of degradation enzymes [3-9]. Abnormal regulation of NAD^+^ metabolism may result in or is associated with serious metabolic disorders and diseases, such as diabetes, cancers, neurological disorders and cardiovascular disease [2,10-17]. Furthermore, the disruption of NAD^+^ synthesis can cause growth suppression and cell death [18-21].

**Figure 1:**
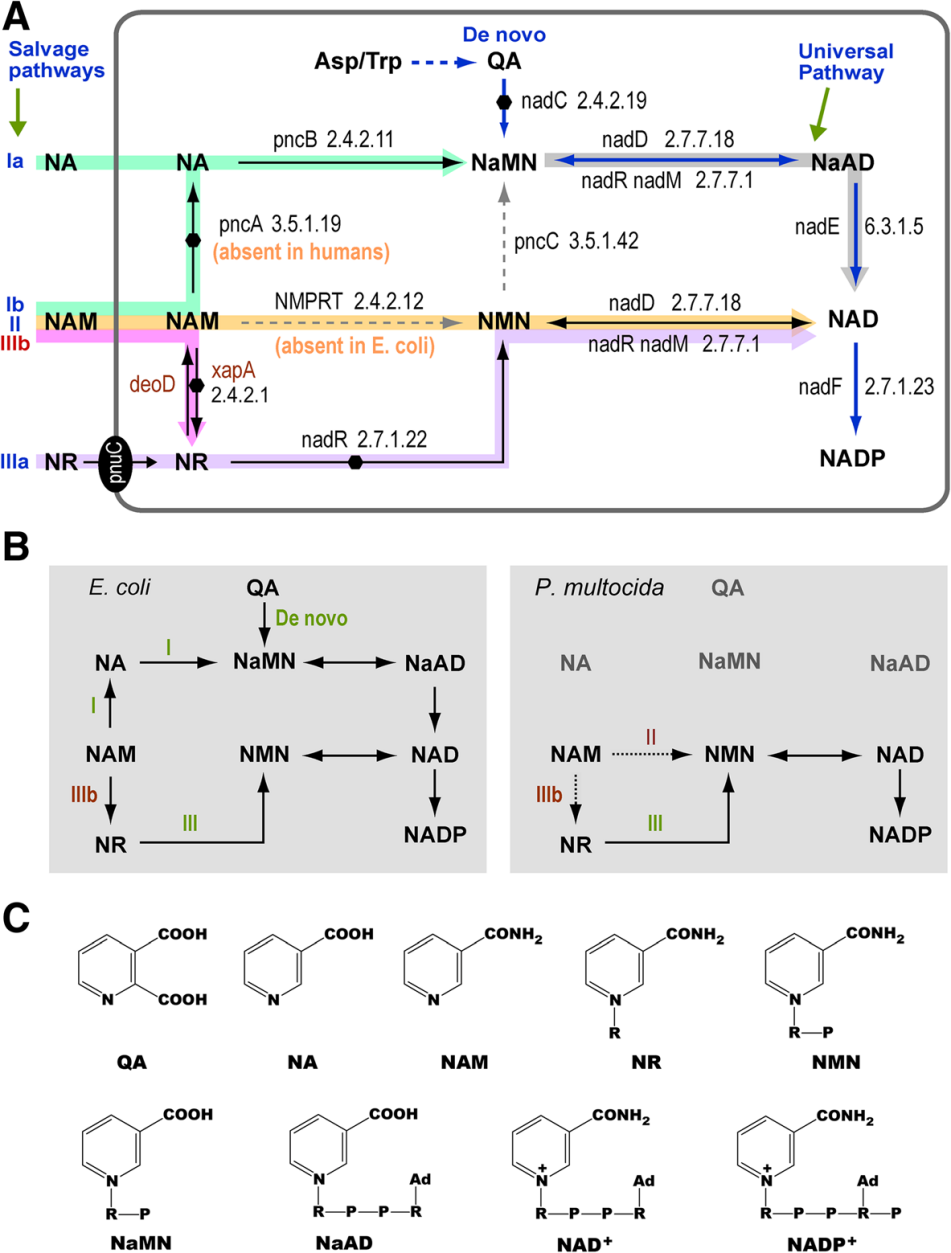
**Illustration of NAD**^**+ **^**synthetic pathways. A)** NAD^+^*de novo* synthetic and salvage pathways in *Escherichia coli*. Dots indicate gene deletions generated by mutagenesis on the pathway. **B)** Comparison of NAD^+^ synthetic pathways between *E. coli* that is able to synthesize NAD^+^ via *de novo* and salvage pathways **I** and **III** and pathogenic bacterium *Pasteurella multocida* that is potentially capable of synthesizing NAD^+^ via salvage pathway **II** and **III**. The xapA/PNP-mediated pathway **IIIb** may enable *P. multocida* and similar pathogenic bacteria to use NAM as a precursor for NAD^+^ biosynthesis. **C)** Chemical structures of NAD^+^ and relevant intermediates (R = Ribose sugar, P = Phosphoric acid, Ad = Adenine). **Abbreviations of compounds:** NA, nicotinic acid; NaAD, nicotinic acid adenine dinucleotide (Deamino-NAD); NAD^+^, nicotinamide adenine dinucleotide; NAM, nicotinamide; NaMN, nicotinic acid mononucleotide; NMN, nicotinamide mononucleotide; NR, nicotinamide riboside; QA, quinolinic acid; **Abbreviations of enzymes:** nadD, NaMNAT, nicotinic acid mononucleotide adenylyltransferase; nadE, NADS, NAD^+^ synthase; nadF, NAD^+^ kinase; nadR/nadM, nicotinamide-nucleotide adenylyltransferase (NMNAT); NMPRT, nicotinamide phosphoribosyltransferase; NRK, ribosylnicotinamide kinase; pncA, nicotinamidase; pncB, NAPRTase, nicotinic acid phosphoribosyltransferase; pncC, NMN deamidase; nadC, QAPRTase, quinolinic acid phosphoribosyltransferase.

NAD^+^ can be synthesized *de novo* from simple amino acid precursors such as tryptophan or aspartate, or converted from intermediates such as nicotinamide (NAM), nicotinic acid (NA) or nicotinamide riboside (NR) via salvage pathways, which are designated as salvage pathways **I** (i.e., (NAM→) NA → NaMN [nicotinic acid mononucleotide] → deNAD [deamino-NAD] → NAD^+^), **II** (i.e., NAM → NMN [nicotinamide mononucleotide] → NAD^+^), and **III** (i.e., NR → NMN → NAD^+^), respectively (Figure 1A) [1,2,12,22-26]. All three pathways are in fact interconnected. However, some organisms (e.g., humans and other vertebrates) may lack a nicotinamidase (pncA; EC 3.5.1.19) to prevent NAM from entering pathway **I**, whereas others (e.g., *Escherichia coli*) lack a nicotinamide phosphoribosyl transferase (NMPRT; EC 2.4.2.12) to prevent NAM from entering pathway **II**[13,27]. In yeast, pathway **I** may be extended by first converting NR to NAM [23].

Some NAD^+^-consuming enzymes may break down NAD^+^ to form various types of ADP-ribosyl groups, in which the NAM moiety is the most common end-product [28,29]. In a variety of physiological events, some of these enzymes (e.g., poly ADP ribose polymerases [PARPs]) can be significantly activated, such as during the regulation of apoptosis, DNA replication, and DNA repair [30], thus potentially leading to the rapid depletion of intracellular NAD^+^, and associated accumulation of NAM [21]. Since NAM is also known as a strong inhibitor of several NAD(P)^+^-consuming enzymes, uncontrolled NAM accumulation may negatively affect not only NAD^+^ metabolism, but also cellular functions such as gene silencing, Hst1-mediated transcriptional repression, and life span of cells [31-34]. Therefore, NAD^+^ salvage pathways **I** and **II** are important not only in regenerating NAD^+^, but also in preventing the accumulation of NAM. While NAM may be converted to NMN by NMPRT (pathway **II**) or NA by pncA (pathway **I**), there were no reports on other enzymes that might act on NAM.

In the present study, we have discovered by genetic and biochemical approaches that xanthosine phosphorylase (xapA; also known as purine nucleoside phosphorylase II [PNP-II], EC 2.4.2.1) is also capable of converting NAM to NR in *E. coli.* XapA was originally identified from *E. coli*, and known to catalyze the reversible ribosyltransfer on purine nucleosides including xanthosine, inosine and guanosine [35-37]. Our data has not only assigned a novel function to xapA, but also uncovered a potential new route in the NAD^+^ salvage, in which the pathway **III** is extended by using NAM as an alternative precursor in xapA-possessing organisms.

## Results

### Genetic disruption of NAD^+^*de novo* biosynthesis and NAD^+^ salvage pathway I in *Escherichia coli*

**Figure 2:**
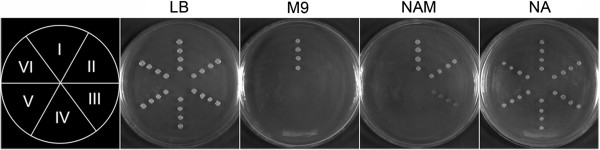
**Growth of wild-type *Escherichia coli *(BW25113) and mutants in LB or M9 agar plates supplied with NAM or NA.** Strains in area I-VI represent BW25113, BW25113Δ*nadC*, BW25113Δ*nadC*Δ*pncA*, BW25113Δ*nadC*Δ*pncA*Δ*xapA*, BW25113Δ*nadC*Δ*pncA*Δ*nadR* and BW25113Δ*nadC*Δ*pncA*Δ*xapA*Δ*nadR*, respectively. See Figure 1 for abbreviations.

In an effort to uncover the new function of *E. coli* xapA in NAD^+^ salvage pathway from nicotinamide, we produced a set of gene knockout mutants deficient in previously defined NAD^+^ synthetic pathways, including NAD^+^*de novo* and NAD^+^ salvage pathways **I** and **III** for genetic investigation purpose (see Table 1, Additional file 1: Figure S1 and Additional file 2: Table S1). We first generated a mutant strain deficient in NAD^+^*de novo* pathway (BW25113Δ*nadC*) that was unable to survive in the M9 minimal medium, but could restore the growth to a level comparable to the wild-type BW25113 when NA or NAM was supplied to allow NAD^+^ synthesized via NAD^+^ salvage pathway **I** (Figure 2 and Table 2).

**Table 1:** ***Escherichia coli *** **strains and plasmids used in this study**

**Strains or plasmids**	**Genotypes and comments**	**Source or reference**
Strain		
DH5α	Routine cloning host	In-house collection
BW25113	rrnB3 Δ*lacZ4787 hsdR514* Δ*(araBAD)567* Δ*(rhaBAD)568 rph-1*	CGSC^*^
BW25113Δ*nadC*	BW25113 with chromosomal *nadC* deletion	This study
BW25113Δ*nadC*Δ*pncA*	BW25113 with chromosomal *nadC* and *pncA* deletion	This study
BW25113Δ*nadC*Δ*pncA*Δ*xapA*	BW25113 with chromosomal *nadC, pncA*, and *xapA* deletion	This study
BW25113Δ*nadC*Δ*pncA*Δ*nadR*	BW25113 with chromosomal *nadC, pncA*, and *nadR* deletion	This study
BW25113Δ*nadC*Δ*pncA*Δ*xapA*Δ*nadR*	BW25113 with chromosomal *nadC, pncA, xapA* and *nadR* deletion	This study
Plasmid		
pKD13	Gene knockout procedure	CGSC^*^
pKD46	Gene knockout procedure	CGSC^*^
pCP20	Gene knockout procedure	CGSC^*^
pBAD-hisA	*bla*^+^	In-house collection
pBAD-EGFP	pBAD-hisA with *EGFP* gene	This study
pBAD-xapA	pBAD-hisA with *xapA* gene	This study
pET28a	*Kana*^*+*^	In-house collection
pET28-xapA	pET28a with *xapA* gene	This study
pEGFP-N2	Template for PCR amplification of *EGFP* gene	In-house collection

^*^CGSC is the *E. coli* Genetic Stock Center of Yale University.

**Table 2:** **Generation time (minutes) of *Escherichia coli *** strains in different culture media*

**No.**	**Strain**	**Pathway Deficiency**	**Medium M9**		**M9 + NA**		**M9 + NAD**^**+**^		**M9 + NAM**	
			**Expected**	**Observed**	**Expected**	**Observed**	**Expected**	**Observed**	**Expected**	**Observed**
1	BW25113	None	+	65.8	+	49.8	+	50.5	+	49.4
2	Δ*nadC*	dn	–	–	+	49.4	+	49.4	+	53.4
3	Δ*nadC*Δ*pncA*	dn, I	–	–	+	50.3	+	49.2	–	380.8
4	Δ*nadC*Δ*pncA*Δ*xapA*	dn, I	–	–	+	49.2	+	50.0	–	620.4
5	Δ*nadC*Δ*pncA*Δ*xapA*/pBAD-xapA	dn, I	–	NT	+	NT	+	+	–	376.4
6	Δ*nadC*Δ*pncA*Δ*xapA*/pBAD-EGFP	dn, I	–	NT	+	NT	+	+	–	626.8
7	Δ*nadC*Δ*pncA*Δ*nadR*	dn, I, III	–	–	+	51.1	+	NT	–	–
8	Δ*nadC*Δ*pncA*Δ*xapA*Δ*nadR*	dn, I, III	–	–	+	49.7	+	NT	–	–

*Notes: NA, nicotinic acid; NAM, nicotinamide; NAD^+^, nicotinamide adenine dinucleotide; NT, not tested; –, No proliferation; +, proliferation; dn, *de novo* NAD^+^ synthesis; I, NAD^+^ salvage pathway **I**; III, NAD^+^ salvage pathway **III**.

We then generated double-deletion mutant BW25113Δ*nadC*Δ*pncA* to also interrupt the conversion from NAM to NA in NAD^+^ salvage pathway **I**. This mutant was expected to only survive in the absence of NA, but not NAM due to the lack of NAD^+^ salvage pathway **II** in *E. coli* (Figure 1). The growth of BW25113Δ*nadC*Δ*pncA* mutant in the absence of NA was confirmed as expected, but we also unexpectedly observed its survival in M9/NAM medium, albeit with a much slower growth rate (i.e., 380.8 min generation time vs. 53.4 min for BW25113Δ*nadC* mutant) (Table 2 and Figure 2). This result suggested the presence of another unknown salvage pathway can participate in the conversion of NAM from medium into NAD^+^.

### Genetic evidence on the involvement of xapA in NAD^+^ salvage pathway

**Figure 3:**
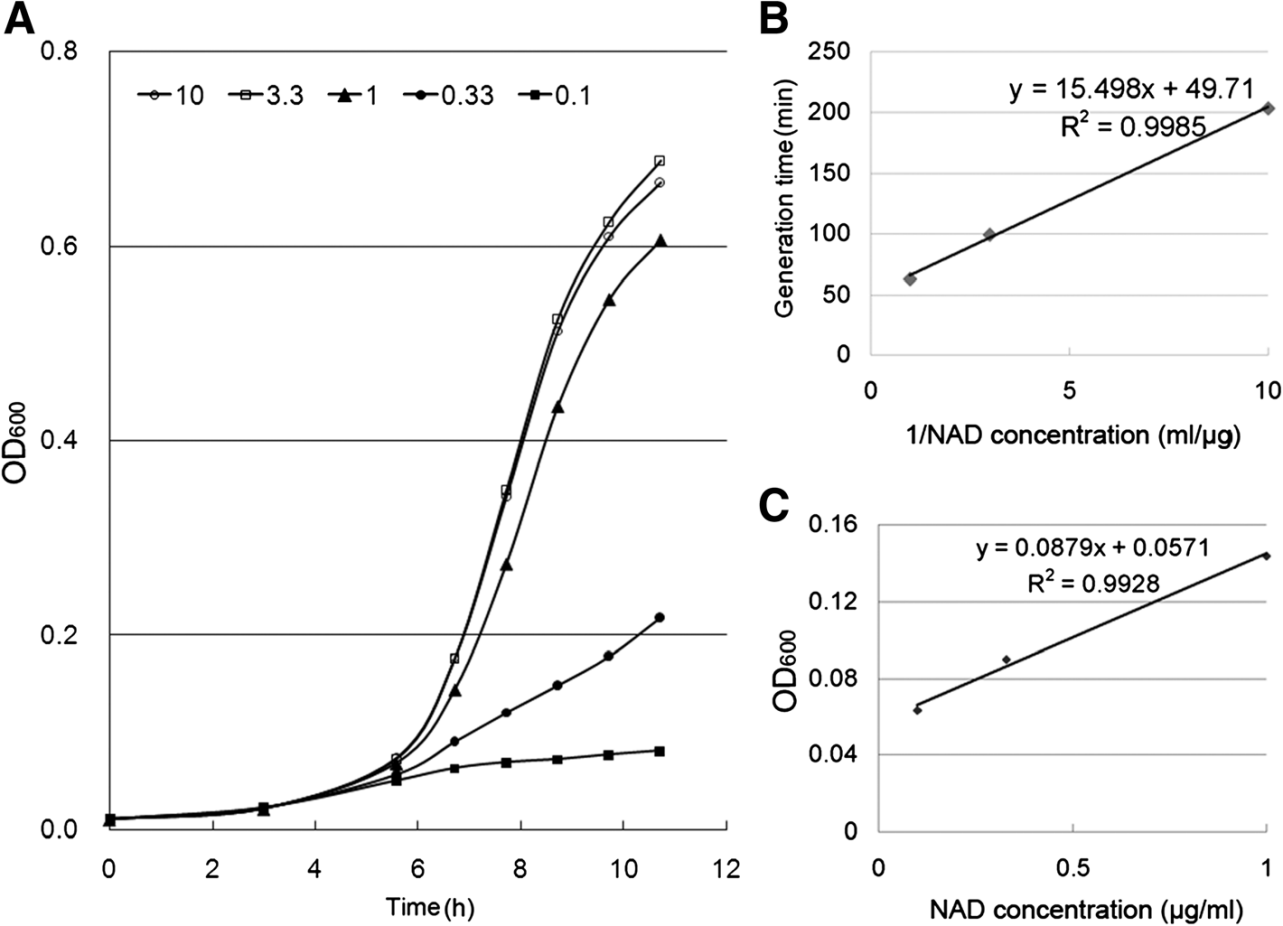
**Dose-dependent effects of NAD**^**+ **^**on the growth of *Escherichia coli *mutant with triple-deletion (BW25113Δ*nadC*Δ*pncA*Δ*xapA*). A)** Growth curve of the mutant in M9 minimal medium supplied with various concentration of NAD^+^. **B)** The relationship of the inverse of the NAD^+^ concentration (from 0.1 to 1 μg/ml) to the bacterial generation time in M9/NAD^+^ medium for 7 h. **C)** The relationship of the NAD^+^ concentration (from 0.1 to 1 μg/ml) to the OD_600_ of the mutant grown in M9/NAD^+^ medium for 7 h.

The ability for BW25113Δ*nadC*Δ*pncA* to grow in M9/NAM medium implied a previously undefined enzyme(s) might be involved in feeding NAM into the NAD^+^ synthesis. The poor efficiency in utilizing NAM was indicative of the presence of an enzyme that might use NAM as an atypical substrate, but the activity was sufficient for bacterial growth when other NAD^+^ intermediates were unavailable. Based on the substrate preference of xapA towards purine nucleosides and the fact that its sister enzyme deoD (PNP-I) is able to use NR as a non-typical substrate to form NAM *in vitro*[38], we hypothesized that xapA might be a candidate enzyme responsible for converting NAM to NR. To test this hypothesis, we developed three multiple gene deletion mutants, namely, BW25113Δ*nadC*Δ*pncA*Δ*xapA*, BW25113Δ*nadC*Δ*pncA*Δ*nadR*, and BW25113Δ*nadC*Δ*pncA*Δ*xapA*Δ*nadR* (Table 1). Among them, the growth of BW25113Δ*nadC*Δ*pncA*Δ*xapA* was worse than that of BW25113Δ*nadC*Δ*pncA* in the M9/NAM medium (i.e., 620.4 min generation time in BW25113Δ*nadC*Δ*pncA*Δ*xapA* vs. 380.8 min in BW25113Δ*nadC*Δ*pncA*) (Figure 2 and Table 2). When a complementary plasmid pBAD-xapA (but not the control vector pBAD-EGFP) was reintroduced into this triple-deletion mutant, its growth rate was restored to a similar level of that of BW25113Δ*nadC*Δ*pncA* (Table 2). We also assessed the growth of this triple-deletion strain in M9/NAD^+^ medium, and observed its normal growth in a dose-dependent manner (Figure 3). The potential involvement of other unknown pathway(s) in making NAD^+^ could be ruled out, since this triple-deletion transformed with pBAD-xapA was unable to growth in the M9 minimal medium (Table 2).

The contribution of xapA in NAD^+^ salvaging was further tested by generating mutants with additional deletion of *nadR* (i.e., BW25113Δ*nadC*Δ*pncA*Δ*nadR* and BW25113Δ*nadC*Δ*pncA*Δ*xapA*Δ*nadR*). Both mutants were able to grow in M9/NA medium, but not in M9 or M9/NAM medium (Figure 2 and Table 2), indicating that NR produced by xapA from NAM was connected to the nadR-mediated NAD^+^ salvage pathway **III**. Collectively, these observations implied the capability for xapA to use NAM as a less efficient substrate to produce NR that could be routed into the pathway **III** (i.e., NAM → NR → NMN → NAD^+^) *in vivo*.

### Biochemical evidence on the conversion of NR from NAM by *E. coli* xapA

**Figure 4:**
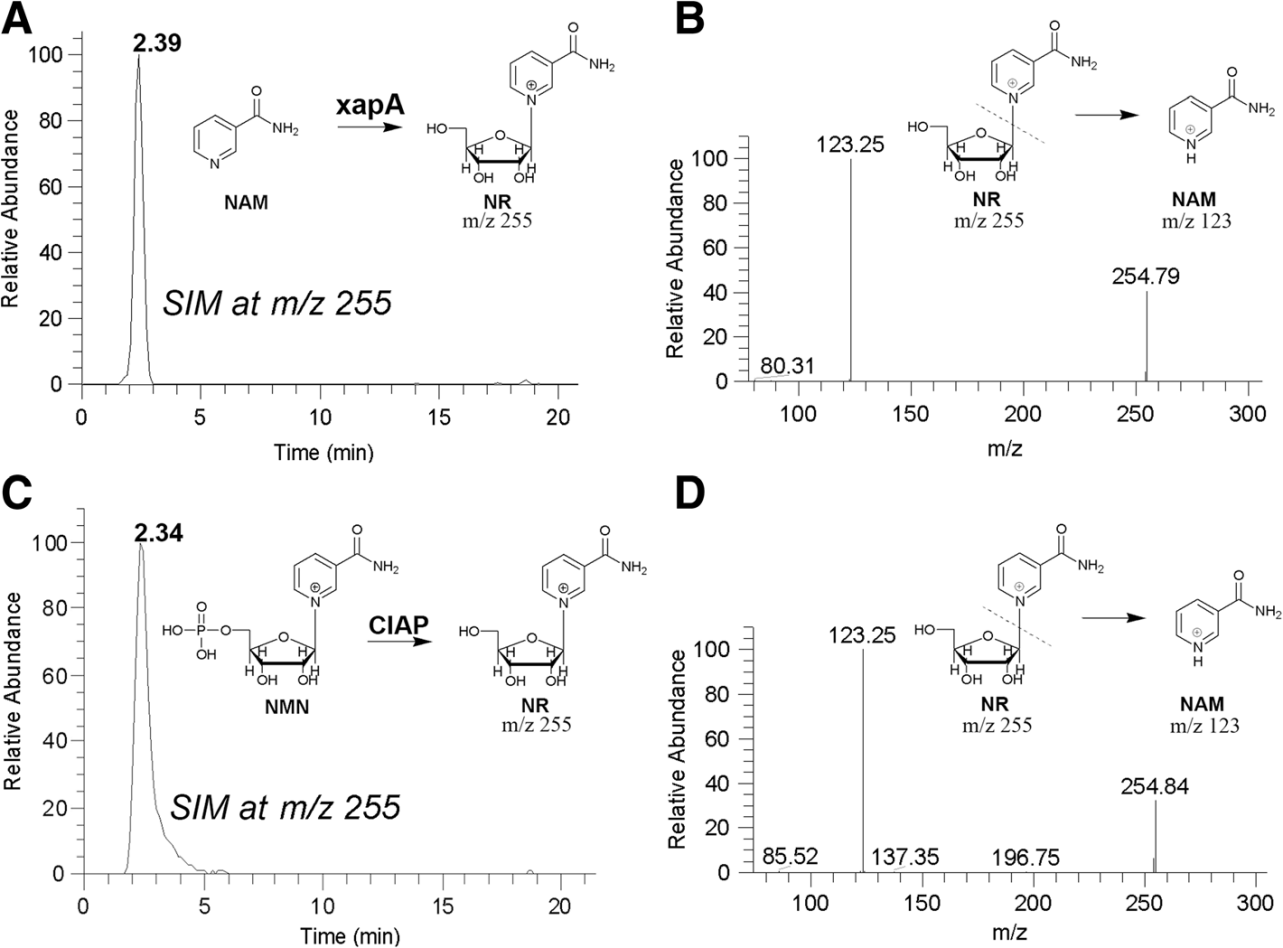
**Biochemical evidence on the synthesis of NR from NAM catalyzed by *E. coli *xapA as determined by HPLC-ESI-MS/MS. A)** Selected-ion monitoring (SIM) chromatogram at m/z 254.3-255.3 Da of NR converted from NAM by recombinant xapA. **B)** Positive ESI-MS/MS spectrum of the NR peak produced by xapA and eluted from HPLC showing an ion fragmentation pattern characteristic to NR, including two major peaks representing NR and the NAM moiety with m/z at 255 and 123, respectively. **C)** SIM chromatogram of NR converted from NAM by CIAP as positive control. **D)** Positive ESI-MS/MS spectrum of the NR peak produced by CIAP and eluted from HPLC. The retention time and MS/MS spectra were identical to those produced by standard NR control, confirming the synthesis of NR from NAM by xapA. See Figure 1 for abbreviations.

The genetic data on the involvement of xapA in converting NAM to NR was further validated by biochemical assays using recombinant xapA protein that was expressed using an *E. coli* expression system and purified into homogeneity (see Additional file 1: Figure S2). Standard NR sample used in these assays was prepared by a hydrolysis of 5′-phosphate groups from NMN by CIAP. The ability for xapA to convert NAM to NR was first confirmed by HPLC-ESI-MS/MS assay. In reactions catalyzed by recombinant xapA and CIAP (positive control), selected-ion monitoring chromatogram (SIM) detected a single peak at the retention time corresponding to NR (Figure 4A and 4C). Further positive MS/MS analysis at m/z 255 detected two major peaks with m/z at 255 and 123, representing NR (255 Da) and the NAM (123 Da) moiety, respectively (Figure 4B and 4D), which confirmed the xapA-catalyzed production of NR from NAM.

Further kinetic analysis showed that the ***K***_**m**_ value towards NAM was 5.81 mM, and the ***V***_**max**_ was at 400 nmol/min/mg protein. The kinetic data indicated that xapA in *E. coli* was much less efficient in using NAM to synthesize NR than using typical substrate (***K***_**m**_ at 5.81 mM on NAM vs. 72 μM on xanthosine) [37], or when compared with other NAD^+^ salvaging enzymes (e.g., ***K***_**m**_ values at 70 μM and 2 μM for pncA and pncB on NAM and NA, respectively) [39,40], but similar to those of deoD (PNP-I) from calf and *E. coli* (i.e., 1.48 mM and 0.62 mM, respectively) in converting the non-typical substrate NR to NAM [38].

**Figure 5:**
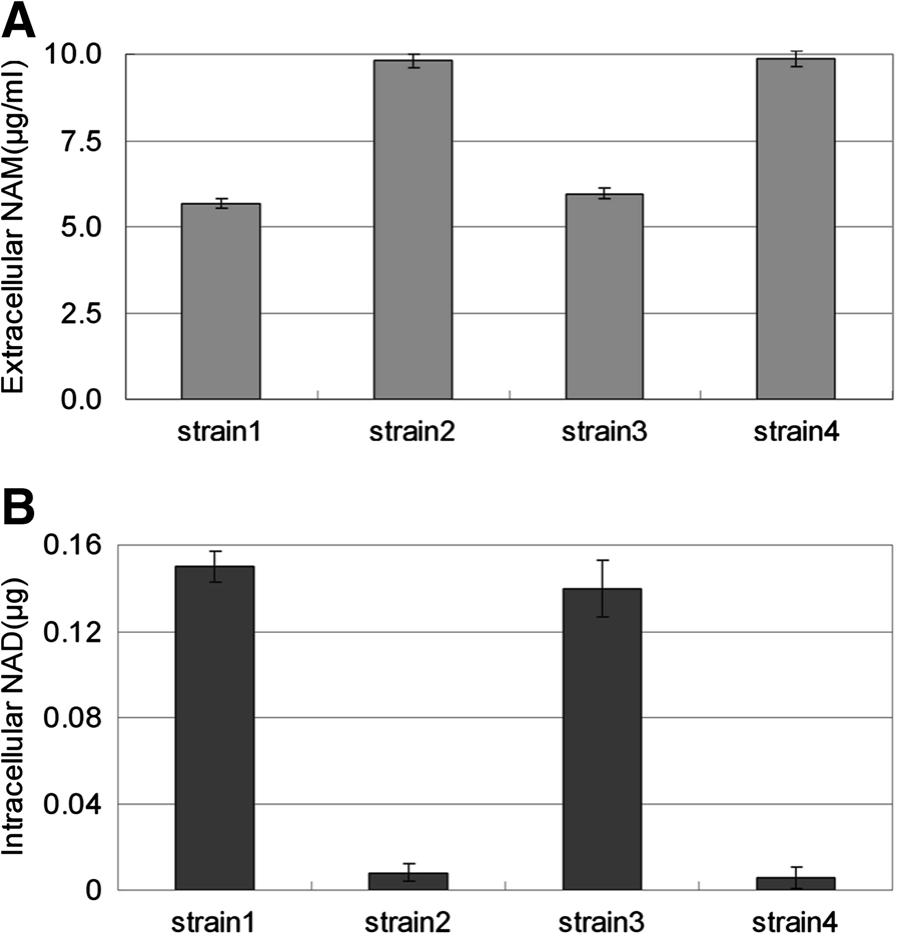
**Consumption of extracellular NAM (A) to form intracellular NAD**^**+ **^**(B) by four strains of *Escherichia coli *derived from BW25113 cultured in M9/NAM medium until the strain BW25113Δ*nadC*Δ*pncA *reached the mid-log phase.** Strain 1, BW25113Δ*nadC*Δ*pncA*; strain 2, BW25113Δ*nadC*Δ*pncA*Δ*xapA*; strain 3, BW25113Δ*nadC*Δ*pncA*Δ*xapA*/pBAD-xapA; and strain 4, BW25113Δ*nadC*Δ*pncA*Δ*xapA*/pBAD-EGFP.

The contribution of xapA in NAD^+^ salvaging was also confirmed in bacterial mutants cultured in M9/NAM medium, in which the consumption of extracellular NAM by the triple-deletion (BW25113Δ*nadC*Δ*pncA*Δ*xapA*) was reduced by 95% in comparison to that by the double-deletion BW25113Δ*nadC*Δ*pncA* (Figure 5A). The consumption of extracellular NAM was restored when vector expressing xapA (but not the EGFP control) was reintroduced to the triple-deletion (Figure 5A). The level of intracellular NAD^+^ was detectable in BW25113Δ*nadC*Δ*pncA* (150 ng), but virtually undetectable in BW25113Δ*nadC*Δ*pncA*Δ*xapA* (Figure 5B). Again, the intracellular NAD^+^ level could be restored by reintroducing xapA into the triple-deletion, but not by EGFP (Figure 5B).

## Discussion

### Contribution of xapA to an alternative NAD^+^ salvage pathway from NAM

Xanthosine phosphorylase (xapA, EC 2.4.2.1) is a second purine nucleoside phosphorylase (PNP-II) in *E. coli*. Similar to PNP-I (deoD), it mainly functions in the purine metabolism by carrying out both phosphorylation and synthesis of purine and purine deoxy-/ribonucleosides [41]. Here we first obtained genetic evidence that xapA was probably involved in NAD^+^ salvage in *E. coli*. We also provided more direct biochemical evidences that xapA was able to synthesize NR from NAM. Both bacterial growth experiments and enzyme kinetic data indicated that xapA used NAM in a much less efficient way than using its typical substrates (i.e., purine analogs), suggesting that NAM served only as a non-typical substrate, which was comparable to the PNP-I. Therefore, the capability to convert NAM to NR appeared to be a “side effect” for xapA. However, such a side-effect was sufficient to maintain the survival of *E. coli* by feeding NAM into the salvage pathway **III** when all other NAD^+^ synthetic pathways were unavailable and only NAM was present in the minimal medium. For clarity, we designated the xapA-mediated extension of NAD^+^ salvage pathway **III** as the salvage pathway **IIIb** (i.e., NAM → NR → NMN → NAD^+^) (Figure 1).

### Potential uses of xapA-mediated salvage pathway in drug development

The true biological function of pathway **IIIb** may be less significant in *E. coli*, as this bacterium is able to synthesize NAD^+^ via multiple routes (i.e., *de novo*, NAD^+^ salvage pathways **I** and **III**). However, we speculate that it may be highly significant for some other pathogenic bacteria that lack NAD^+^*de novo*, NAD^+^ salvage pathway **I** and/or **II** for NAD^+^ synthesis. One of the examples might be the gram-negative coccobacillus *Pasteurella multocida* that causes a range of diseases in humans and animals. It appears to be V-factor-independent, indicating its capability to utilize NAM as the pyridine nucleotide, as well as NAD^+^, NMN and NR to synthesize NAD^+^[42]. Analysis of NAD^+^ biosynthesis pathways reveals that *P. multocida* lacks NAD^+^*de novo* and NAD^+^ salvage pathway **I** but possesses NAD^+^ salvage pathway **II** and NAD^+^ salvage pathway **III** for the presence of nadV, NMPRT homolog in bacteria, and nadR [26] (Figure 1B). Furthermore, a *PNP* homologue (see Additional file 3: Text S1) is also present in the *P. multocida* genome. Accordingly, it seems reasonable to speculate that *P. multocida* may synthesize NAD^+^ from NAM through NAD^+^ salvage pathway **II** and/or NAD^+^ salvage pathway **IIIb**. However, the hypothesis on the potential contribution of NAD^+^ salvage pathway **IIIb** to NAD^+^ biosynthesis in such bacteria remains to be tested. If the hypothesis is confirmed, the xapA or its isoenzyme(s) may be explored as a novel target for developing therapeutics.

In fact, the NAD^+^ salvage pathways of human is similar to that of *P. multocida* in that humans lack NAD^+^ salvage pathway **I**, but possess NMPRT-mediated NAD^+^ salvage pathway **II** and NRK (isozyme of nadR)-mediated NAD^+^ salvage pathway **III** (Figure 1A) [23,24,43]. NMPRT is highly expressed in many types of tumor cells, including human hematologic malignancies, to maintain adequate levels of NAD^+^[44-46]. Inhibitor(s) of NMPRT, such as FK866, has been in Phase II clinical trials [47,48]. However, NAM was found to have an antidote potential for the cellular effects of FK866 [49], which indicates that the NAD^+^ synthesis pathways from NAM may be not completely disrupted. As the PNP-mediated new salvage pathway is also present in mammals (see Additional file 2: Table S2 and Additional file 3: Text S2), it remains to be tested whether human PNP (counterpart of xapA) is also able to utilize NAM to synthesize NR as an alternative to pathway **II** (i.e., via pathway **IIIb**), thus responsible for the slow anti-cancer action of FK866. In fact, the enzymes involved in the pathway **IIIb**, such as human PNP and NRK, are all effective anticancer drug targets [50,51]. When the inhibitors of PNP and/or NRK are used in combination with FK866, rapid NAD^+^ depletion and NAM accumulation may occur, hopefully increase the anticancer efficacy or widen the antitumor spectra, or even conquer the drug resistance.

### Contribution of xapA to a new pyridine nucleotide cycle

Additionally, this newly discovered pathway **IIIb** may also be significant in the pyridine nucleotide cycles (PNCs) that are mediated by the breakdown and re-synthesis of NAD^+^[52]. PNCs are economic and efficient approaches to recycle NAD^+^ intermediates back into NAD^+^ without the actual consumption of NAD^+^, which ensures the homeostatic balance between NAD^+^ degradation and replenishment. Thus far, PNCs are found to consist of three to seven reaction steps, which are correspondingly named as PNC III–VII (see Additional file 1: Figure S3) [52]. When NAD^+^ is broken down to NAM by the NAD^+^-consuming enzymes, the NAM-based NAD^+^ re-synthetic pathways involved in PNCs are identical to the NAD^+^ salvage pathways. More specifically, the salvage pathway **I** and **II** are the same as the NAD^+^ resynthesis routes of PNC V and PNC III, respectively. Therefore, the presence of xapA-mediated NAD^+^ salvage pathway **IIIb** would also extend the related PNC IV, which is proposed here as PNC IV-B to distinguish it from the existing PCN-IV cycle (see Additional file 1: Figure S3).

## Conclusions

We have provided genetic and biochemical evidences showing that xanthosine phosphorylase (xapA) in *E. coli* is able to utilize nicotinamide (NAM) as an atypical substrate to synthesize nicotinamide riboside (NR), which extends the NAD^+^ salvage pathway **III** to use NR as an alternative precursor (i.e., pathway **IIIb**). This unexpected discovery not only assigns a new function to xapA, but also increases our current knowledge on the NAD^+^ biosynthesis and pyridine nucleotide cycles.

## Methods

### Bacterial strains, plasmids, media and reagents

The BW25113 strain of *E. coli* served as a parent strain for generating mutants with single to multiple gene deletions within the NAD^+^ synthetic pathways (see Table 1 for a list of strains and plasmids used in this study). Bacteria were cultured in lysogeny broth (LB), LB agar, M9 broth or M9 agar as described [53]. When required, supplements were typically used at the following final concentrations: 100 μg/ml of ampicillin, 50 μg/ml of kanamycin, 1 mmol/liter of L-arabinose, 10 μg/ml of NAM, 10 μg/ml of NA, and 10 μg/ml of NAD^+^. All chemicals were purchased from Sigma-Aldrich (St. Louis, MO) with purity at ≥99%. NAM was further purified with high-performance liquid chromatography (HPLC) to remove minor contaminating NA.

### Genetic construction of various NAD^+^ synthesis pathway-deficient mutants

A series of *E. coli* mutants with single to multiple gene deletions in the NAD^+^*de novo* and salvage pathways were constructed from the wild-type BW25113 stain using a λ Red-mediated recombination system as described (Table 1) [53,54]. Briefly, PCR products were generated from template plasmids carrying a kanamycin resistant (Km^R^) gene flanked by FLP-recognition target sites using primers with 36-nucleotide extensions that were homologous to regions adjacent to the genes to be inactivated and (see Additional file 2: Table S3 for primer sequences). The chromosomal genes were replaced by the corresponding PCR products via the λ Red-mediated recombination system. The resulting Km^R^ colonies were selected and verified by PCR and sequencing of the PCR products, and the kanamycin resistant cassette was removed by introducing pCP20 helper plasmid that carried the yeast Flp recombinase and ampicillin resistant gene (Amp^R^). The Red and FLP helper plasmids were subsequently cured by growth at 37°C because they are temperature-sensitive replicons.

### Phenotypic determination of NAD^+^ synthesis deficiency by selective media

The phenotypic deficiencies of mutants were validated by their capabilities to utilize different precursors to synthesize NAD^+^ in various selective media. All strains were washed twice in M9 minimum medium to remove trace amounts of nutrients and resuspended in specified selective media. For plate growth assay, 0.2 μl suspensions of the *E. coli* strains (OD_600_ = 0.1) were dotted onto agar plates containing M9 alone or M9 plus either NA or NAM. Plates were incubated at 37°C for 12 h or longer. For determining growth rates, strains were diluted in specified liquid media (OD_600_ = 0.005), cultured at 37°C and OD_600_ values were measured every hour as described [53]. The generation times (T_d_) were calculated during the exponential phase of growth according to the formula: T_d_ = (t_2_-t_1_) × {log(2)/[log(q_2_/q_1_)]}, where t_1_ and t_2_ represented times, and q_1_ and q_2_ represented the number of cells at t_1_ and t_2_, respectively. Additionally, the dose-dependent effect of NAD^+^ on the triple-deletion strain (BW25113Δ*nadC*Δ*pncA*Δ*xapA*) was measured in M9 medium containing NAD^+^ at various concentrations (i.e., 0, 0.1, 0.33, 1, 3.3, and 10 μg/ml). The growth rate and generation time of this mutant were determined as described above.

### Genetic validation on the involvement of xapA in NAD^+^ salvage pathway

To further validate the involvement of xapA in NAD^**+**^ salvage pathway, a genetic complementation experiment was performed by reintroducing *xapA* into the triple-deletion mutant (BW25113Δ*nadC*Δ*pncA*Δ*xapA*). The *xapA* ORF was amplified and reconstructed into pBAD-hisA at the *Eco*RI and *Xho*I sites. The same pBAD-hisA vector carrying the enhanced green fluorescence protein (EGFP) gene (pBAD-EGFP) was constructed as control. The plasmids were then transformed into the BW25113Δ*nadC*Δ*pncA*Δ*xapA* strain. Transformed cells were cultured on LB plates containing ampicillin, and the positive clones were selected for growth phenotypic examination. The growth rates of the transformed cells in M9/NAM medium were determined as described above.

### Cloning, expression and purification of recombinant *E. coli* xapA

The open reading frame (ORF) of *xapA* was amplified by PCR (see Additional file 2: Table S3 for primer sequences) from *E. coli* genomic DNA using the high-fidelity *Pfu* DNA polymerase, and cloned into pET28a expression vector (Novagen) flanking the *Eco*RI and *Hind*III sites as described [53]. The resulting pET28-xapA was sequenced to ensure the absence of undesired mutations. For expressing fusion proteins, the Rosetta (DE3) strain of *E. coli* transformed with pET28-xapA was grown at 37°C with constant shaking until OD_600_ reached to 0.8. After adding 0.1 mM isopropyl β-D-1-thiogalactopyranoside (IPTG) into the media to induce protein expression, bacteria were allowed to grow for 8 h at 16°C and harvested by centrifugation. Cell pellets were stored at -80°C, or immediately resuspended in lysis buffer, followed by the purification of soluble xapA proteins using the QIA express Ni-NTA Protein Purification System according to the manufacturer’s protocol (Qiagen, Hilden, Germany). Purified protein was washed with phosphate buffered saline (PBS, pH 7.4) and concentrated by ultrafiltration membrane with a molecular weight cutoff (MWCO) at 10 kDa. The protein purity was generally greater than 99% as evaluated by SDS-PAGE (see Additional file 1: Figure S2).

### Enzyme assays for xapA activity

The activity for xapA to convert NAM to NR was assayed similarly as described [55]. Briefly, the reaction (100 μL volume) was performed in 50 mM MES buffer (pH 6.0) containing 10 μg xapA protein, 1 mM NAM and 1 mM ribose-1-phosphate (R1P) at 37ºC for 60 min. In the meantime, a positive control used calf intestinal alkaline phosphatase (CIAP, 1000 U) (Sigma) to convert NMN (12.4 mg) to NR under the same reaction condition to validate the detection of NR [24]. Reactions were stopped by chilling on ice.

The product NR was determined by HPLC-electrospray ionization tandem mass spectrometry (HPLC-ESI-MS/MS) using an Agilent 1200 HPLC system coupled with a Thermo Finnigan LCQ Deca XP Electrospray Ion Trap Mass Spectrometer (Thermo Quest-Finnigan Co., San Jose, CA) [56]. Briefly, HPLC used a reversed-phase Venusil XBP C18 column (100 mm Length × 2.1 mm i.d., 5 μm) (Agela Technologies, China). The mobile phase was composed of 5 mM ammnonium formate (A) and methanol (B) with the linear gradient elution: 0–10 min, A from 98% to 90% and B from 2% to 10%; 10–15 min, A from 90% to 30% and B from 10% to 70%. The mobile phase was then returned to 98% A at 15.1 min, and the column was re-equilibrated with 98% A for 7 min. Other settings include: constant flow rate at 0.25 ml/min; injection volume at 5 μl; ESI-MS spray voltage at 5.5 kV, and the capillary voltage at -15.0 V, and capillary temperature at 285°C. Nitrogen was used as both the sheath gas and auxiliary gas at 50 and 5 units, respectively. Helium was used as the collision gas in MS/MS. Multiple positive scanning modes were cyclically alternated during the analyses in a data-dependent fashion as follows: 1) the full first scan event was operated in a range of *m/z* from 110 – 2,000 Da; 2) the selected ion monitoring (SIM) scans were set at *m/z* 254.8 for NR, *m/z* 123.0 for NAM, and *m/z* 334.8 for NMN; and 3) the MS/MS scans were set at 254.8@cid 18 for NR, 123.0@cid 30.0 for NAM, and 334.8@cid 19 for NMN. The isolation width was set to 1.0 Da, and the ejected ions were detected by the electron multiplier with a gain at 5 × 10^5^. Data were analyzed by Xcalibur Software version 1.4 (Thermo Scientific).

Kinetic parameters for xapA enzyme were determined by measuring the decreased absorbance of NAM at 262 nm with a Synergy H1 microplate reader (BioTek, USA) as described [55]. The reaction was performed in 50 mM MES buffer (pH 6.0) containing 20 mM R1P, 0.1 mg/ml xapA protein and varied concentrations of NAM at 37°C for 30 min. Michaelis-Menten plots and the linear transformations (Lineweaver-Burk, Hanes-Woolf and Eadie-Hofstee) were used for determining the kinetic parameters.

### Quantitative analysis of NAD^+^ synthesis on the xapA-mediated NAD^+^ salvage pathway from NAM

We also directly tested the utilization of NAM by xapA in the bacterial mutants by measuring their consumption of extracellular NAM and the production of NAD^+^ in cells. In this experiment, four mutants (i.e., BW25113Δ*nadC*Δ*pncA,* BW25113Δ*nadC*Δ*pncA*Δ*xapA*, BW25113Δ*nadC*Δ*pncA*Δ*xapA/*pBAD-xapA and BW25113Δ*nadC*Δ*pncA*Δ*xapA/*pBAD-EGFP) were cultured in the M9/NAM medium. The cultures were maintained until the BW25113Δ*nadC*Δ*pncA* strain reached the mid-log phase. A volume of the bacterial suspensions containing approximately 1 × 10^9^ BW25113Δ*nadC*Δ*pncA* cells was collected by centrifugation at 15,000 ×g for 10 min. Equal volumes of the other three strains were also collected. After centrifugations, bacterial pellets and supernatants were separately collected. The supernatants were freeze-dried for measuring extracellular NAM. The pellets were resuspended in 2 ml of deionized water and ultrasonicated for 10 min. After centrifugation at 15,000 ×g for 15 min at 4°C, supernatants were collected and freeze-dried for measuring intracellular NAD^+^. The concentrations of NAD^+^ and NAM were determined by HPLC-ESI-MS as described above.

### Statistical analysis

All experiments were performed independently for at least three times. Statistically significant differences were calculated by two-tailed Student’s *t*-test using SPSS software (version 19.0) (http://www-01.ibm.com/software/analytics/spss/).

## Competing interests

The authors declare that they have no competing interests.

## Authors’ contributions

WRD, GZ, JZS designed the experiments; WRD, CCS performed the experiments including *E. coli* mutagenesis assay, bacterial growth analysis, recombinant protein studies; WRD, SHH carried out xapA enzyme assays; SHH performed NAM and NAD^+^ detection; WRD, GZ wrote the manuscript; GZ, LXX, JZS reviewed and edited the manuscript. All authors read and approved the final manuscript.

## Supplementary Material

Additional file 1: Figure S1 PCR verification of gene deletions in the *E. coli* mutants. ST1-ST6 represents BW25113, BW25113Δ*nadC*, BW25113Δ*nadC*Δ*pncA*, BW25113Δ*nadC*Δ*pncA*Δ*xapA*, BW25113Δ*nadC*Δ*pncA*Δ*nadR* and BW25113Δ*nadC*Δ*pncA*Δ*xapA*Δ*nadR*. **Figure S2**. SDS-PAGE (12%) analysis of recombinant xapA protein expressed in *E. coli*. Lanes 1: protein marker; lane 2: cell-free extract before induction with IPTG; lane 3: cell-free extract after IPTG induction; lane 4: recombinant xapA protein. **Figure S3**. Potential contribution of xapA-mediated conversion from NAM to NR (marked by an asterisk) in the pyridine nucleoside cycles (PNCs). Pathways unique to *E. coli* or vertebrates are marked. Click here for file

Additional file 2: Table S1 The expected product sizes (bp) for PCR of the four specified genes in different strains used in the study. **Table S2**. The presence of nicotinamide riboside kinase (NRK) gene and purine nucleoside phosphorylase (PNPase) gene in vertebrates. **Table S3**. List of primers and applications. Click here for file

Additional file 3: Text S1 Protein sequence of predicted purine nucleoside phosphorylase (PNPase) in *Pasteurella multocida*. **Text S2**. Protein sequences of nicotinamide riboside kinase (NRK) and purine nucleoside phosphorylase (PNPase) in vertebrates. Click here for file
