# Supplementary material for: New function for Escherichia coli xanthosine phophorylase (xapA): genetic and biochemical evidences on its participation in NAD+ salvage from nicotinamide
Source: BMC Microbiol. 2014 Feb 8;14:29. doi: 10.1186/1471-2180-14-29 (PMC3923242; doi:10.1186/1471-2180-14-29)
Supplement: Additional file 1: Figure S1 — PCR verification of gene deletions in the E. coli mutants. ST1-ST6 represents BW25113, BW25113ΔnadC, BW25113ΔnadCΔpncA, BW25113ΔnadCΔpncAΔxapA, BW25113ΔnadCΔpncAΔnadR and BW25113ΔnadCΔpncAΔxapAΔnadR. Figure S2. SDS-PAGE (12%) analysis of recombinant xapA protein expressed in E. coli. Lanes 1: protein marker; lane 2: cell-free extract before induction with IPTG; lane 3: cell-free extract after IPTG induction; lane 4: recombinant xapA protein. Figure S3. Potential contribution of xapA-mediated conversion from NAM to NR (marked by an asterisk) in the pyridine nucleoside cycles (PNCs). Pathways unique to E. coli or vertebrates are marked. [file 1471-2180-14-29-S1.doc]

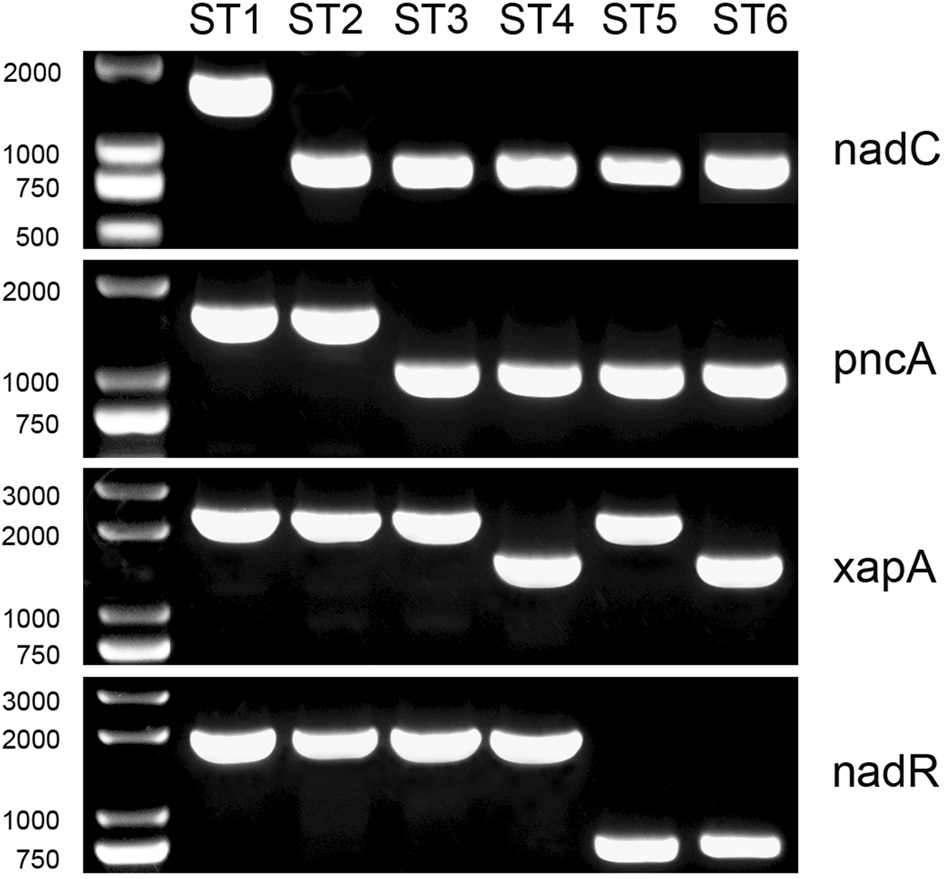


**Figure S1.** PCR verification of gene deletions in the *E. coli* mutants. ST1-ST6 represents BW25113, BW25113Δ*nadC*, BW25113Δ*nadC*Δ*pncA*, BW25113Δ*nadC*Δ*pncA*Δ*xapA*, BW25113Δ*nadC*Δ*pncA*Δ*nadR* and BW25113Δ*nadC*Δ*pncA*Δ*xapA*Δ*nadR*.


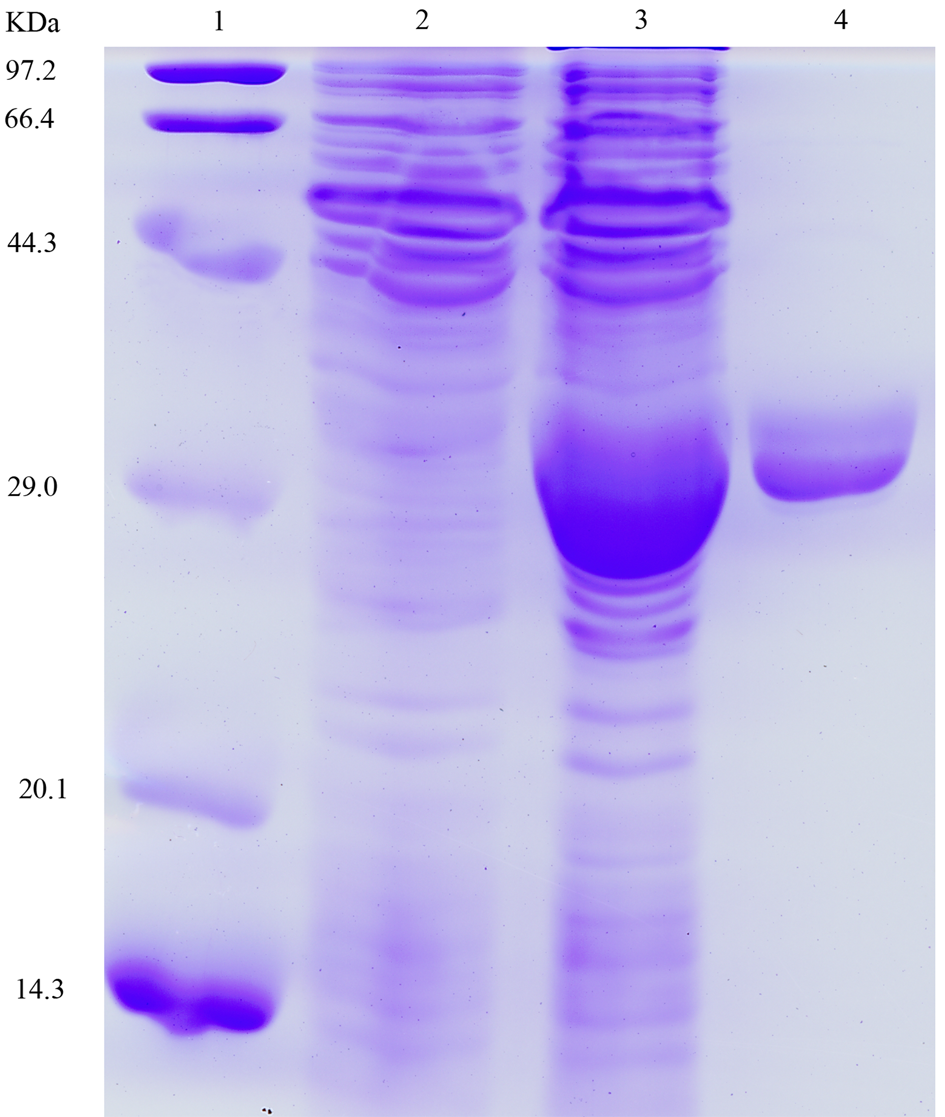


**Figure S2**. SDS-PAGE (12%) analysis of recombinant xapA protein expressed in E. coli. Lanes 1: protein marker; lane 2: cell-free extract before induction with IPTG; lane 3: cell-free extract after IPTG induction; lane 4: recombinant xapA protein.


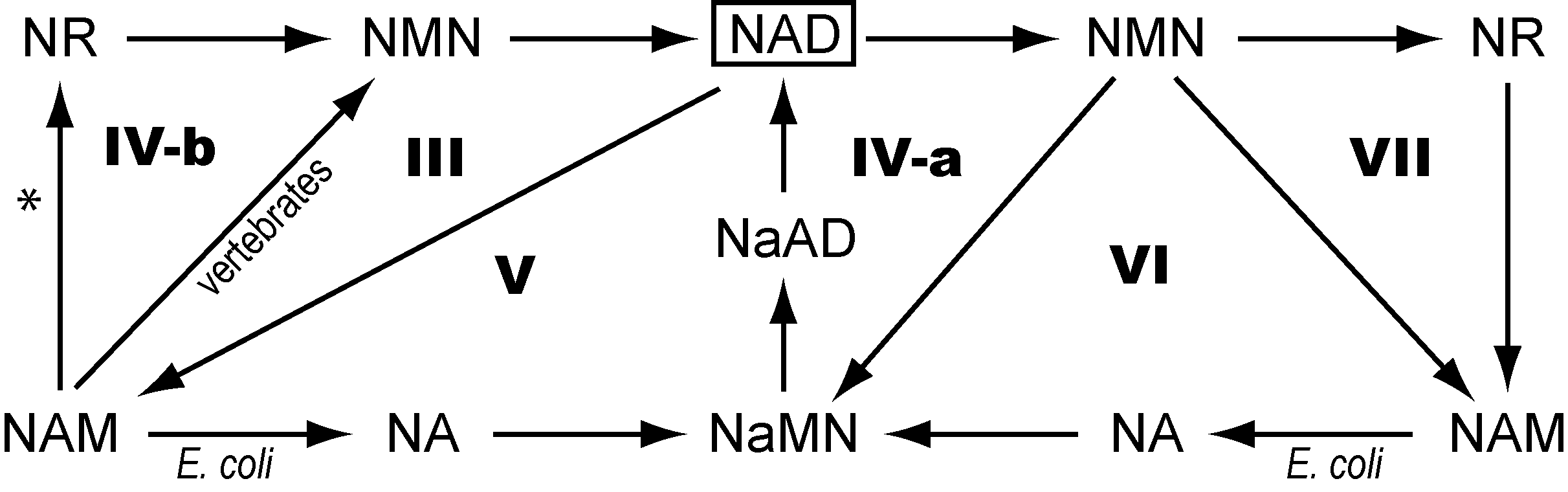


**Figure S3.** Potential contribution of xapA-mediated conversion from NAM to NR (marked by an asterisk) in the pyridine nucleoside cycles (PNCs). Pathways unique to *E. coli* or vertebrates are marked.
