# Supplementary material for: New function for Escherichia coli xanthosine phophorylase (xapA): genetic and biochemical evidences on its participation in NAD+ salvage from nicotinamide
Source: BMC Microbiol. 2014 Feb 8;14:29. doi: 10.1186/1471-2180-14-29 (PMC3923242; doi:10.1186/1471-2180-14-29)
Supplement: Additional file 2: Table S1 — The expected product sizes (bp) for PCR of the four specified genes in different strains used in the study. Table S2. The presence of nicotinamide riboside kinase (NRK) gene and purine nucleoside phosphorylase (PNPase) gene in vertebrates. Table S3. List of primers and applications. [file 1471-2180-14-29-S2.doc]

**Table S1.** The expected product sizes (bp) for PCR of the four specified genes in different strains used in the study.

|  | strain1 | strain2 | strain3 | strain4 | strain5 | strain6 |
| --- | --- | --- | --- | --- | --- | --- |
| nadC | 1662 | 873 | 873 | 873 | 873 | 873 |
| pncA | 1582 | 1582 | 1045 | 1045 | 1045 | 1045 |
| xapA | 2250 | 2250 | 2250 | 1521 | 2250 | 1521 |
| nadR | 1864 | 1864 | 1864 | 1864 | 715 | 715 |

Strain1-strain6 represents BW25113, BW25113Δ*nadC*, BW25113Δ*nadC*Δ*pncA*, BW25113Δ*nadC*Δ*pncA*Δ*xapA*, BW25113Δ*nadC*Δ*pncA*Δ*nadR* and BW25113Δ*nadC*Δ*pncA*Δ*xapA*Δ*nadR,* respectively.

**Table S2.** The presence of nicotinamide riboside kinase (NRK) and purine nucleoside phosphorylase (PNPase) genes in vertebrates.

|  | NRK | PNPase | NMNAT | PNC IV-B |
| --- | --- | --- | --- | --- |
| Mammal | √ | √ | √ | √ |
| Bird | √ | √ | √ | √ |
| Reptile | √ | √ | √ | √ |
| Amphibian | √ | √ | √ | √ |
| Fish | √ | √ | √ | √ |

**Table S3.** List of primers and applications.

| Primer | Sequences(5’-3’) | Comments |
| --- | --- | --- |
| nadC-F | GTAGCATGTTTCTACCTTATGATTCGTTAGCTATCTGGAGTTTTAACATGATTCCGGGGATCCGTCGACC | Knockout |
| nadC-R | CGCGATGGAGCGGATAAATCTGTCAACTATTAGCGAAAACGCATTGAAAGTGTAGGCTGGAGCTGCTTCG | Knockout |
| nadC-U | GAATCAAACAGTGAGCGGAGACG | Knockout test |
| nadC-D | CATAGCGGGTGGTGGTAGGC | Knockout test |
| pncA-F | CCAAAACCTGCGCGGCGAACTGACGCCGGATGATTAAGGAGACTGTAATGATTCCGGGGATCCGTCGACC | Knockout |
| pncA-R | CCAGTACAGAATTGTAGGCCAGCGTAAAATTACCCCTGTGTCTCTTCCCATGTAGGCTGGAGCTGCTTCG | Knockout |
| pncA-U | GCGTAGTGACGATTTATCCAGG | Knockout test |
| pncA-D | TGACCGCTGTTATTATGATTGG | Knockout test |
| xapA-F | TCGCTATAAAAAGGCGTTAGATTCCACCCTACAGAAAAAGGATATGTATGATTCCGGGGATCCGTCGACC | Knockout |
| xapA-R | ACCCGCGAGGCCGTTTCTTATTGTTTTTCTCAGGCAATTTTGCGCAGAAATGTAGGCTGGAGCTGCTTCG | Knockout |
| xapA-U | CCGTTGTCGCCAGTGTAATAGG | Knockout test |
| xapA-D | CAGCACGAAGGCATCCAGACC | Knockout test |
| nadR-F | GGCGTTAAAAAACTCTCCGACGCGCTTAGCGTGTTCGACGACTTATAATGATTCCGGGGATCCGTCGACC | Knockout |
| nadR-R | TACCTCGCCTTTGAGCAGTTTCATCACGGTTATCTCTGCTCCCCCATCATTGTAGGCTGGAGCTGCTTCG | Knockout |
| nadR-U | GGTATGGGAAGGAACGCGTCC | Knockout test |
| nadR-D | CCTCAATTTCGTGGCGGGTC | Knockout test |
| EGFP-F | CGGGGTACCATACCATGGTGAGCAAGGGC | Expression |
| EGFP-R | CCGGAATTCTTACTTGTACAGCTCGTCC | Expression |
| pBAD-xapA-F | CCGCTCGAGTCTCAGGTTCAATTTTCTC | Expression |
| pBAD-xapA-R | CCGGAATTCCTCAGGCAATTTTGCGCAG | Expression |
| pET28-xapA-F | GAATTCATGTCTCAGGTTC | Expression |
| pET28-xapA-R | AAGCTTTCAGGCAATTTTGCGCAG | Expression |
