# Supplementary material for: New function for Escherichia coli xanthosine phophorylase (xapA): genetic and biochemical evidences on its participation in NAD+ salvage from nicotinamide
Source: BMC Microbiol. 2014 Feb 8;14:29. doi: 10.1186/1471-2180-14-29 (PMC3923242; doi:10.1186/1471-2180-14-29)
Supplement: Additional file 3: Text S1 — Protein sequence of predicted purine nucleoside phosphorylase (PNPase) in Pasteurella multocida. Text S2. Protein sequences of nicotinamide riboside kinase (NRK) and purine nucleoside phosphorylase (PNPase) in vertebrates. [file 1471-2180-14-29-S3.doc]

**Text S1**. Protein sequence of predicted purine nucleoside phosphorylase (PNPase) in *Pasteurella multocida*.

>gi|15603156|ref|NP_246228.1| purine nucleoside phosphorylase [Pasteurella multocida subsp. multocida str. Pm70]

MTPHINAPAGAFADVVLMPGDPLRAKYIAETFLQDVKEITNVRNMLGFTGTYKGRKISVMGHGMGIPSCSIYTKELITEYGVKKIIRVGSCGAVRMDVKLRDVVIGFGACTDSKVNRIRFKNHDFAAIADFDMTMAAVQAAKAKGLNVHVGNLFSADLFYTPDVEMFDVMEKYGILGVEMEAAGIYGVAAEFGAKALTICTVSDHIRTHEQTTPEERQLTFNDMIEIALESVLIGDNA

**Text S2**. Protein sequences of nicotinamide riboside kinase (NRK) and purine nucleoside phosphorylase (PNPase) in vertebrates.

NRKs in vertebrates

>gi|8923530|ref|NP_060351.1| nicotinamide riboside kinase 1 isoform 1 [Homo sapiens]

MKTFIIGISGVTNSGKTTLAKNLQKHLPNCSVISQDDFFKPESEIETDKNGFLQYDVLEALNMEKMMSAISCWMESARHSVVSTDQESAEEIPILIIEGFLLFNYKPLDTIWNRSYFLTIPYEECKRRRSTRVYQPPDSPGYFDGHVWPMYLKYRQEMQDITWEVVYLDGTKSEEDLFLQVYEDLIQELAKQKCLQVTA

>gi|21703978|ref|NP_663472.1| nicotinamide riboside kinase 1 [Mus musculus]

MKRFVIGIGGVTNGGKTTLAKSLQKHLPNCSVISQDDFFKPESEIDIDENGFLQYDVLEALNMEKMMSAVSCWMENPGSSAGPAALESAQGVPILIIEGFLLFNYKPLDTIWNRSYFLTVPYEECKRRRSTRVYEPPDPPGYFDGHVWPMYLKHRQEMSSITWDIVYLDGTRSEEDLFSQVYEDVKQELEKQNGL

>gi|118104090|ref|XP_424839.2| nicotinamide riboside kinase 1 [Gallus gallus]

MKILVIGLGGVTNGGKTTLAEKLKKLLPNCDTLCQDDFFKPESEVETDERGFKLYDVLDALYMDEMVKTIHNWMKSHTSSGVATEEPMDTCNSLKMTEDVYILIVEGFLLYNYEPLNELWNRRYFLTLPYEECKRRRSTRVYQPADTPGYFDGHVWPMYLKYKNEMEESASNIVYLDGTKSQEELLSYVYNDIIQELKKLREGNQQVTA

>gi|327263548|ref|XP_003216581.1| nicotinamide riboside kinase 1-like [Anolis carolinensis]

MKILVIGLGGVTNGGKTTLSRKLKDQFPNCTIISQDDFFKPESEVAIDDNGFLQYDVLDALYMEKMVMSIRSWMSNPEDSVLTRPPERTHVGQKRAEEIHILIVEGFLLYNYKPLSDIWDKKYFLTIPYEECKRRRSKRIYNPPDPPGYFDGHVWPMYLKHKKEMEENETGIVYLDGMQSQEKLYSRVFNDIAHEMEKAGA

>gi|147903169|ref|NP_001084826.1| nicotinamide riboside kinase [Xenopus laevis]

MKQFIIGISGITNGGKTTLANRLLKVLPNCTLICQDDYFKPDSYIETDENGFKQFDVIEALDMETMMAAVHSWIKQSQDTLTVDENKEMHHACEKKAYFLIVEGFLLYHYKPLENVLNRKYFLIIPYEESKKRRSQRIYNPPDPPGYFDGHVWPMYLKHKKEMEEANNEIVYLDGTKSEEEIQSSVYADIINSFSVHKESY

>gi|156718010|ref|NP_001096547.1| nicotinamide riboside kinase [Xenopus (Silurana) tropicalis]

MKQFIIGISGITNGGKTTLANRLLKLLPNCSLICQDDYFKPDSDIETDENGFKQYDIIEALDMETMIKAVHSWIKLSQDVLAMEEKKEMCSTCEEKAYFLIVEGFLLYHYKPLENVLNRKYFLSIPYEESKQRRSRRIYNPPDPPGYFDGHVWPMFLKHKKEMEETHSDIVYLDGTKSEDEIQSLVYSDIISSFSIHK

>gi|169159087|emb|CAQ15022.1| nicotinamide riboside kinase [Danio rerio]

MKFIIGIGGVTNGGKTTLTGRLIKNLPNCCVVHQDDFFKPQDQIELGEDGFRQWDVITALDMDAMVNTVKGWMENPVKFARSHGVSVSTTSDPDSDIHILIVEGFLLYNYKPLIDVYNKCFYVTIPYEECKRRRSTRTYTVPDPPGLFDGHVWPMYLKHRTEMENSSLDIQYLDGMSSKDELYNQVYEDIQNSLLNIL

PNPs in vertebrates

>gi|157168362|ref|NP_000261.2| purine nucleoside phosphorylase [Homo sapiens]

MENGYTYEDYKNTAEWLLSHTKHRPQVAIICGSGLGGLTDKLTQAQIFDYGEIPNFPRSTVPGHAGRLVFGFLNGRACVMMQGRFHMYEGYPLWKVTFPVRVFHLLGVDTLVVTNAAGGLNPKFEVGDIMLIRDHINLPGFSGQNPLRGPNDERFGDRFPAMSDAYDRTMRQRALSTWKQMGEQRELQEGTYVMVAGPSFETVAECRVLQKLGADAVGMSTVPEVIVARHCGLRVFGFSLITNKVIMDYESLEKANHEEVLAAGKQAAQKLEQFVSILMASIPLPDKAS

>gi|7305395|ref|NP_038660.1| purine nucleoside phosphorylase [Mus musculus]

MENEFTYEDYETTAKWLLQHTEYRPQVAVICGSGLGGLTAHLKEAQIFDYNEIPNFPQSTVQGHAGRLVFGLLNGRCCVMMQGRFHMYEGYSLSKVTFPVRVFHLLGVETLVVTNAAGGLNPNFEVGDIMLIRDHINLPGFCGQNPLRGPNDERFGVRFPAMSDAYDRDMRQKAFSAWKQMGEQRKLQEGTYVMLAGPNFETVAESRLLKMLGADAVGMSTVPEVIVARHCGLRVFGFSLITNKVVMDYENLEKANHMEVLDAGKAAAQTLERFVSILMESIPLPDRGS

>gi|363738060|ref|XP_003641950.1| purine nucleoside phosphorylase-like [Gallus gallus]

MAYAEEDRNSYEVYKETADWLRARTARCPKIAIVCGSGLGDLADMLEHKMVFPYEDIPHFPRSTVSGHAGRLVFGELSGRPCVCMQGRFHFYEGYSISRITFPIRVFFLLGVEILIVTNAAGGLNPHFQVGDVMLIRDHISMFGMGGQNPLRGPNDERFGVRFPCMSDAYDQDLLSLAMESAQELGFLGFTRDGVYCMMAGPCYETIAECRMLQALGADAVGMSTVPEVIVARHCGLCVLGLSLITNTAVMSYGSQEKASHEDVLRVSACQAKALQKLVVHLISKLGPNSP

>gi|327278334|ref|XP_003223917.1|purine nucleoside phosphorylase-like [Anolis carolinensis]

MDSKREERYTYEDVKKTADWLLSKTKHRPKIAIICGSGLGGLADLLKDQVAFEYSKIPNFPQSTVVGHAGRLVFGNLSGAPCVVMQGRFHMYEGYPLWKVTFPVRIFHLLGVETLIVTNAAGGLNPDYKVGDIMVIRDHINMPGFAGQNPLMGPNDERFGARFPAMSDAYDQDVRKLAHTVASEMGCSGCVREGVYVALGGPNYETIAECRFLQRLGADAVGMSTVPEVIVARHCGIRVFGFSLITNKAILDYETKEKANHAEVLEASRQSARTLEKLVSLMVQRIERNNNVA

>gi|55742254|ref|NP_001006720.1| purine nucleoside phosphorylase [Xenopus (Silurana) tropicalis]

MSPPAPAGQKQEEERHTYEDYKQTADWLLSKTKHRPTVAIVCGSGLGGLGNLLTEPDAFNYSDIPNFPQSTVPGHAGRLIFGNLSGKPCVCMQGRFHFYEGYPLWKVTFPVRVFRLMGVEVIIVTNAAGGLNQEFSVGDIMVIKDHINMLGFAGQNPLFGHNDERFGPRFPPMSDAYDKEMRSLLLATGKELGFNNMREGVYCGIGGPNFETIAECRYLSKIGADAVGMSTVHEVVVARHCGLRSLGISLITNKAVMDYDSKATANHEEVLQAGRDSAKYMEKLVSTFLQHLNLNQV

>gi|147898869|ref|NP_001079809.1| purine nucleoside phosphorylase [Xenopus laevis]

MSEKESCTYEEYKQTSDWLLSKTKHRPIVAIVCGSGLGGLGELLKDQQAFNYCDIPNFPKSTVPGHAGRLIFGNLSGKPCVCMQGRFHFYEGYPLWKVTFPVRVFHLMGVEAIILTNAAGGLNQEFSVGDIMVIKDHINMVGFAGQNPLFGHNEDRFGPRFPPMSDAYNKNMRSLLLAAGKELGYNNMREGVYCGLGGPNFETIAECRFLNKLGADAVGMSTVHEVVVARHCGLRILGISLITNKAYGL

>gi|239052020|ref|NP_991218.2| nucleoside phosphorylase-like [Danio rerio]

MSTSSECSFSYEEYKETADWLLANTDIRPKVAIICGSGLGGLADLLDNKQVFSYDKIPRFPHSTVQGHKGQLVFGELNGKQCVCMQGRFHFYEGYNVATVTYPVRVFFLLGIETLIVTNAAGGLNPKFKVGDIMVIKDHINMPGFAGQNPLCGHNEERFGVRFPCMSDAYDRDLAQLVRKTAKELGCDSFLQEGVYCMLAGPSYETIAECRVLQMLGADAVGMSTVPEVVIARHCGIRVFGLSLITNKVVTDYDSKERANHEEVLETTRMRTEDLQRIVSNVVRKM
